# Sialic Acid-Targeted Biointerface Materials and Bio-Applications

**DOI:** 10.3390/polym9070249

**Published:** 2017-06-27

**Authors:** Yuting Xiong, Minmin Li, Qi Lu, Guangyan Qing, Taolei Sun

**Affiliations:** 1 State Key Laboratory of Advanced Technology for Materials Synthesis and Processing, Wuhan University of Technology, 122 Luoshi Road, Wuhan 430070, China; xiongyuting6567@whut.edu.cn (Y.X.); zjtxlq2005@whut.edu.cn (Q.L.); 2 College of Mechanical Engineering, Jiangxi University of Technology, 115 Ziyang Road, Nanchang 330098, China; liminmin@whut.edu.cn; 3 School of Chemistry, Chemical Engineering and Life Science, Wuhan University of Technology, 122 Luoshi Road, Wuhan 430070, China

**Keywords:** sialic acids, biointerface materials, cancer detection and imaging, drug delivery

## Abstract

Sialic acids (SAs) are typically found as terminal monosaccharides attached to cell surface glycoconjugates, which play crucial roles in various biological processes, and aberrant sialylation is closely associated with many diseases, particularly cancers. As SAs are overexpressed in tumor-associated glycoproteins, the recognition and specific binding of SA are crucial for monitoring, analyzing and controlling cancer cells, which would have a considerable impact on diagnostic and therapeutic application. However, both effective and selective recognition of SA on the cancer cell surface remains challenging. In recent years, SA-targeted biointerface materials have attracted great attention in various bio-applications, including cancer detection and imaging, drug delivery for cancer therapy and sialylated glycopeptide separation or enrichment. This review provides an overview of recent advances in SA-targeted biointerface materials and related bio-applications.

## 1. Introduction

**Figure 1:**
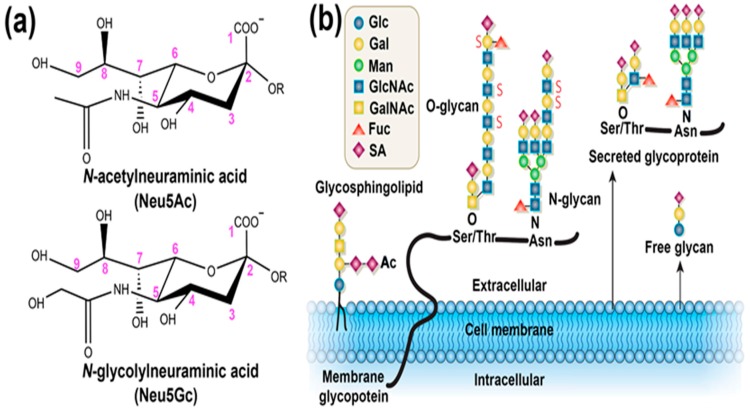
(**a**) The structures shown are two major sialic acids (SAs) found in mammalian cells, *N*-acetylneuraminic acid (Neu5Ac) and *N*-glycolylneuraminic acid (Neu5Gc). SAs share a nine-carbon backbone, a carboxylic acid at the C-1 position, and various α-glycosidic linkages to the underlying sugar chain (R) from the C-2 position. The only difference between the two SAs is the additional oxygen atom in the *N*-glycolyl group of Neu5Gc; (**b**) SAs are typically found at the outer ends of *N*-and *O*-linked glycans attached to the cell surface, or to glycoproteins and glycosphingolipids expressed at the cell surface. (Ac, *O*-acetyl ester; Fuc, fucose; Gal, galactose; GalNAc, *N*-acetylgalactosamine; Glc, glucose; GlcNAc, *N*-acetylglucosamine; Man, mannose; S, sulfate ester.) Adapted with permission [2]. Copyright 2007, Nature Publishing Group.

Since the structure determination in the mid-1950s, important findings and detailed investigations have confirmed the significant role of sialic acids (SAs) in biology and medicine [1]. As a class of nine-carbon backbone monosaccharides, SAs are typically found at the outermost ends of the glycan chains that are attached to glycosylated proteins and lipids (Figure 1) [2,3]. This kind of monosaccharide is very diverse in their structure, and constitutes a family with more than 50 members, in which the most prevalent forms found in mammalian cells are *N*-acetylneuraminic acid (Neu5Ac) and *N*-glycolylneuraminic acid (Neu5Gc). Furthermore, it has been demonstrated that SAs can mediate or modulate a wide variety of physiological and pathological processes, contributing to various cell-cell and cell-pathogen interaction, such as cell adhesion, signal transduction, and immune responses [4]. Given that SAs play crucial roles in various biological, pathological, and immunological processes, some errors produced in their biosynthesis or degradation will inevitably cause dramatic undesirable biological consequences even diseases such as cancer, neurodegenerative disorders, and diabetes mellitus [5]. Of special note is the fact that the overexpression of SAs on the cell membrane surface has been closely associated with the malignant or metastatic phenotypes of various cancers. For example, enhanced expression of terminal α2, 6-linked SAs on *N*-linked glycans and of sialyl-Lewis X on *O*-linked glycans has often been correlated with carcinomas of the brain, colon, breast, and cervix [6,7,8,9]. Increased ST6 Gal I (β-galactoside α2, 6 sialyltransferase) and subsequently elevated levels of cell-surface α2, 6-linked SAs have been associated with metastasis and therapeutic failure in colorectal cancer [10,11]. On the other hand, it has been observed that the total level of free SA and lipid- and protein-bound SA is elevated in the serum of patients with ovarian neoplasia [12], colorectal cancer [13], and oral cancer [14], compared those healthy individuals. Moreover, it has been shown that the evident accumulation of Neu5Gc, a nonhuman SA, from consumption of red meat in human tissues, in particular for liver, could potentially incite inflammation, and develop a much higher incidence of hepatocellular carcinomas [15]. For years, cancer has been one of the major causes of death globally, accounting for 13% of all deaths annually [16]. Thus, recognizing and monitoring cancer cells, and establishing cancer diagnoses are urgently required for early therapy, with the purpose of alleviating pain and increasing the speed of recovery. Current conventional methods are typically based on the detection of tumor-related biomarkers. In this respect, SA generally present in tumor-associated glycoproteins is an ideal molecule target, and monitoring SA expression can provide a reasonable index for early detection and diagnosis of various cancers [17].

Currently, commercially available SA quantification reagents are capable of assessing the SA content on the cell surface, generally through multiple enzymatic and labeling procedures that are costly, time consuming, and often require highly specialized personnel. In addition, some approaches based on lectins and chemical modifications have been developed for SA measurement. To this end, the first critical step is usually the separation of SA from a complex sample through capillary electrophoresis [18] and gas or liquid chromatography, accompanied by a detection step involving mass spectrography [19], colorimetry [20] and fluorometric assay [21]. However, some drawbacks, such as low time and cost efficiency and the need for expensive and complex apparatuses, limit the promotion and application of these approaches. With the booming development of materials science, researchers in this field are cooperating closely with biochemists to make their best efforts to develop artificial materials capable of recognizing and detecting SA molecules. Among which, artificial biointerface materials, especially polymeric biointerfaces, capable of targeting and monitoring of SA through heterogeneous interaction have attracted considerable interests, due to their great potential to design and develop various devices. Such SA-targeted biointerface materials can achieve the facile detection by responding to SA-bearing glycoconjugates, even in cancer cells, in virtue of the optical, electrochemical or hydrophilic-hydrophobic properties of the material itself [22], which have presented broad potential applications related to cancers, such as biodetection and biosensing [23], controllable drug delivery and release [24], and bio-separation [25].

In this review article, we summarize the recent advances of SA-targeted biointerface materials including SA-imprinted polymeric particles, boronic acid-modified surface, polymeric nanocarriers, and bio-inspired polymeric materials. The intriguing bio-applications of these biointerface materials related to cancers are introduced. Finally, some existing challenges and outlooks for the future development of SA-targeted biointerface materials are presented.

## 2. SA-Imprinted Particles for Cancer Cell Imaging

Tailor-made molecular imprinting is an efficient technology to create chemically synthetic receptors predesigned with binding specificity and high affinity toward target molecules based on a templating process at the molecular level [26,27]. First, monomers containing functional groups self-assemble around a template molecule. Then, a polymeric mold around the template forms after the monomers copolymerize with others. Subsequent removal of the template generates three-dimensional binding sites that are complementary to the template in size and shape in the polymer. Hence, SA-imprinted particles can present high binding capacities toward SA.

**Figure 2:**
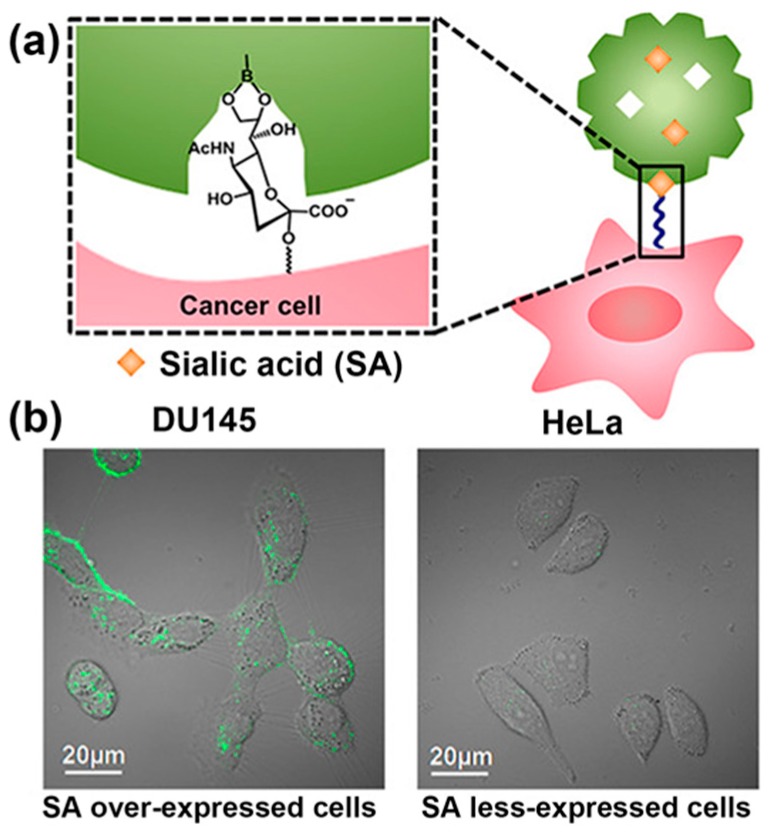
(**a**) Selectivity binding mechanism of SA-imprinted fluorescent conjugated polymeric nanoparticles to cancer cells; (**b**) Confocal laser scanning microscope images of DU 145 (left) and HeLa (right) cells incubated with SA-imprinted fluorescent conjugated polymeric nanoparticles for 24 h at 37 °C. Adapted with permission [31]. Copyright 2017, American Chemical Society (ACS).

Generally, receptors of different monosaccharides can be obtained using the monosaccharide templates conjugated to vinylbenzeneboronic acid, taking advantage of molecular imprinting technology [28]. Based on similar methods, SA-imprinted fluorescent polymers have also been prepared as optical sensors or sensor coatings [29,30]. Recently, several reports on the SA-imprinted fluorescent particles for the selective labeling of SA and imaging of living cells have attracted considerable attention [31,32,33,34,35]. In 2017, Liu et al. synthesized SA-imprinted fluorescent conjugated polymeric nanoparticles (NPs) for application in cancer cell imaging (Figure 2) [31]. First, a fluorescent conjugated backbone poly(fluorine-*alt*-benzothiadiazole) grafted with boronic acid groups was prepared, which could bind to many SA molecules through the interaction between boronic acid and SA. Then, SA molecules were embedded in fluorescent polymeric NPs by a facile reprecipitation process. Afterwards, these SA molecules were removed from the surface of NPs through acidifying the NP solution and a dialysis process, and SA-imprinted fluorescent conjugated polymeric NPs were obtained. As a reference, non-imprinted NPs were also prepared by avoiding the introduction of SA molecules in the reprecipitation process. To assess the selective binding behavior of the as-formed NPs toward SA, DU 145 and HeLa cells, two typical human cancer cell lines, were chosen to perform the cellular imaging experiment. The SA overexpression level was significantly higher in DU 145 cells than in HeLa cells [36,37]. As a result, the non-imprinted NPs failed to differentiate between DU 145 and HeLa cells, whereas, SA-imprinted NPs turned out to be a perfect solution to the problem. The cell imaging assay clearly indicated that SA-imprinted NPs exhibited selective staining for DU 145 cancer cells (Figure 2b). Therefore, these results present an efficient method of recognizing SA overexpression on the cancer cell surface and achieving targeted cancer cell imaging with SA-imprinted NP materials.

**Figure 3:**
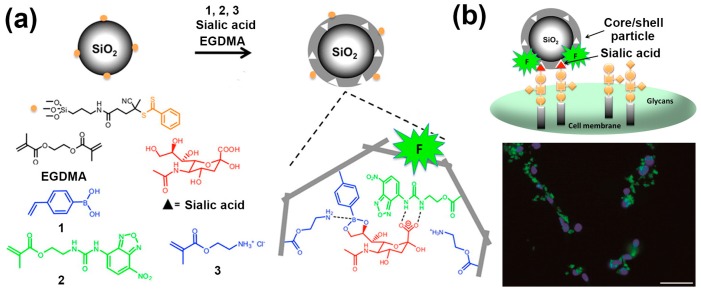
(**a**) Procedure for the grafting of an SA-imprinted shell on silica core particles through a new ternary complex hybrid imprinting approach, and the principle of using SA-imprinted fluorescent core-shell particles as tools for recognizing terminal SA of glycan motifs and imaging of cancer cells; (**b**) Targeting and imaging of cancer cells with SA-imprinted NPs. Adapted with permission [32]. Copyright 2015, ACS.

In addition, Shinde et al. developed SA-imprinted fluorescent core-shell particles for specific labeling of SA on the cell surface [32]. As shown in Figure 3a, imprinting was achieved using a ternary complex hybrid approach combining reversible boronate ester formation between 4-vinylphenylboronic acid (1) and SA, the binary hydrogen bonding of nitrobenzoxadiazole (NBD) fluorophore-appended urea monomers (2) and cationic primary amine monomer (3) with SA carboxylic acid and OH groups. These monomers were grafted from 200 nm core silica particles resulting in a shell thickness of approximately 10 nm. Consequently, the particles displayed a stronger affinity for SA than other reported hosts, such as boronate hosts. With the aid of a strong and specific affinity for SA overexpressed on the cell surface, SA-imprinted fluorescent particles could successfully stain cancer cell lines in cell imaging experiments (Figure 3b). This study provides a novel strategy for designing SA-imprinted fluorescent particles with an ultra-strong affinity for application in cancer cell imaging. Later, using this kind of SA-imprinted fluorescent core-shell particles, they further performed an extended detection of SA expression level of different chronic lymphocytic leukemia cell lines [33]. The results showed that SA-imprinted fluorescent nanoparticles can be used as a plastic antibody to detect SA and even screening different tumor cell of various stages.

Molecular imprinting technology could be easily extended to other monosaccharide-imprinted NPs for more monosaccharide templates. For example, by using different monosaccharides (SA, fucose, or mannose) as the template, Wang et al. and Yin et al. prepared several types of imprinted fluorescent NPs through the boronate affinity oriented surface imprinting approach [35,38]. Depending on different the expression levels of monosaccharides on the surface of various cancer cells, these monosaccharide-imprinted NPs could specially target cancer cells over normal cells, and present a good potential as suitable fluorescent probe for the imaging of different cancer cell types. 

Therefore, the uniform, small size and recognizable bright fluorescence of the SA-imprinted particles with specific SA-binding properties make it a suitable fluorescent probe for targeting cancer cell and in imaging applications.

## 3. Boronic Acid-Modified Surfaces for Detection

Boronic acid and their derivatives, especially phenylboronic acid (PBA), are known to form reversible complexes with cis-diol-containing compounds, such as various monosaccharides. The specific interaction between these compounds has attracted widespread interest for construction of a novel molecular recognition system as a platform for diverse applications, including chromatographic and membrane separation [39], sensing [40], drug delivery, and imaging [41]. Moreover, boronic acids have also been demonstrated to interact with the biological membrane of various cells, viruses, and bacteria with carbohydrate moieties displayed on their surfaces under a physiological pH with high stability [42,43]. Generally, PBA derivatives can form stable complexes with saccharides only in their charged dissociated forms under alkaline conditions, because most PBA derivatives are weak acids possessing a p*K*_a_ of approximately 9 [44,45]. Nevertheless, as an exception, a complex formed between uncharged non-dissociated PBA and SA is still quite stable even at an acidic pH lower than its p*K*_a_ value owing to the special binding modes [46,47]. Given the overexpression of SA on the surfaces of various cancer cell types, interface materials modified with PBA enable the highly specific recognition and detection of SA, and the further identification of cancer.

**Figure 4:**
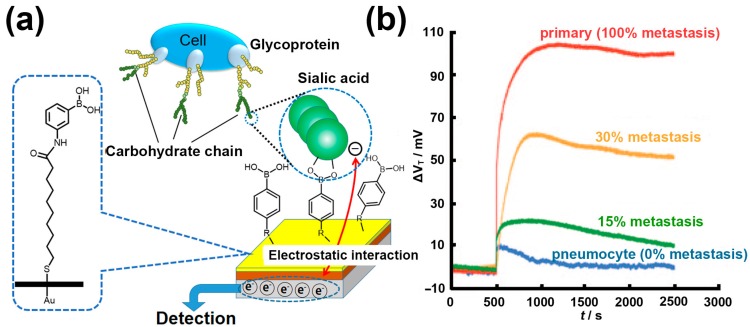
(**a**) Schematic illustration of potentiometric SA and cell detection with a PBA-modified gold electrode; (**b**) Change in the threshold voltage (*V*_T_) of the sensor as a function of time upon the addition of cell suspensions (10^6^ cells mL^−1^) from tumoral lung specimens with various degrees of metastasis. Adapted with permission [49]. Copyright 2010, Wiley Online Library.

In 2009, Matsumoto et al. reported a method for the potentiometric detection of SAs by using the reversible and specific binding of a PBA-based sensor with SA molecules [48]. Specifically, a self-assembled monolayer (SAM) of 10-carboxy-1-decanethiol was first anchored on a gold electrode surface, followed by a condensation reaction resulting in the introduction of PBA onto the SAM terminal and the generation of a PBA-modified gold electrode (Figure 4a). By monitoring changes in the charge density that occur on the gold electrode surface depending on whether SA with a carboxyl anion is captured, this sensor achieved the detection of free SA molecules at a carefully optimized environmental pH value. Furthermore, this PBA-modified gold electrode exhibited its ability of directly capturing glycan SA component present on the cell surface, and differentiating the altered levels of SA expression on different cell surfaces under physiological aqueous conditions. This method without any enzymatic and labeling procedures enabled the label-free, noninvasive, and real-time detection of SA. In order to further verify its application potential, Matsumoto et al. also applied this technique to the assessment of tumor metastasis [49]. The experimental results indicated that this PBA-modified gold electrode could differentiate the degrees of tumor metastasis through the detection of cell-membrane SA (Figure 4b), meaning that it may serve as a straightforward and quantitative approach for the analysis of tumor malignancy and the degree of metastasis during intra- or postoperative diagnosis.

**Figure 5:**
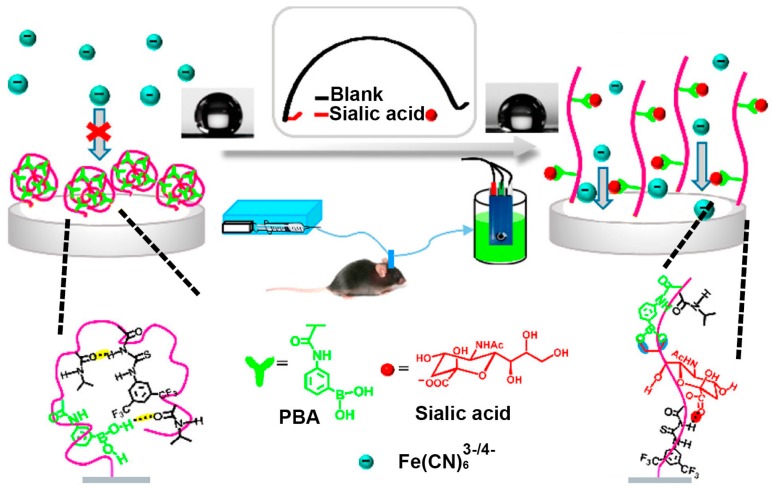
Schematic illustration of the novel phenylboronic acid copolymer-based electrochemical biosensor coupled with in vivo microdialysis for measurement of SA in the brain of a live mouse. The recognition of SA changed the conformation and wettability of the copolymer through synergetic hydrogen-bonding interactions, enabling sensitive electrochemical impedance spectroscopy detection. Adapted with permission [55]. Copyright 2017, ACS.

Currently, many electrochemical cytosensors having the advantages of convenience and simplicity have been developed for disease diagnosis [50,51]. However, most of them require complicated fabrication processes, which result in poor reproducibility and reliability. Recently, Dervisevic et al. reported a novel electrochemical cytosensor based on the single-step electro-polymerization of 3-thienyl boronic acid and thiophen on the graphite electrode surface, which showed highly sensitive detection capacity toward cancer cells by means of the specific interaction between boronic acid groups and SA [52]. Similarly, some electrochemical sensors, mainly based on the PBA-modified electrode capable of recognizing SA with a low detection limit and identifying cancer cells, were also developed. These sensors exhibited broad potential applications in routine SA detection and tumor identification [53,54]. To further improve sensitivity and selectivity in the recognition and monitoring of SA, a multiple-component stimuli-responsive copolymer system bearing PBA, as an electrochemical biosensor for SA detection, was introduced by Ding et al. (Figure 5) [55]. Taking advantage of the “recognition-mediating-function” concept [56,57], the copolymer system was elaborately designed, contained PBA group as the specific recognition unit, phenylthiourea as the mediating unit, and poly(*N*-isopropylacrylamide) as the functional switching unit. Relying on the specific recognition of SA and the amplification of the recognition signal through the conformational transition of stimuli-responsive copolymer, this smart copolymer enabled the access and enrichment of redox labels, and significantly improved the SA detection sensitivity with a detection limit down to 0.4 pM, by the electrochemical impedance spectroscopy method performed in solution containing [Fe(CN)_6_]^3−/4−^. Moreover, such an electrochemical biosensor with remarkable SA detection performance combined with in vivo microdialysis was successfully applied to evaluate the dynamic change of SA levels in the brains of live mice with or without Alzheimer’s disease. The potential of this interface material constituted biosensor in the dynamic monitoring of SA level in vivo should enable it to study the role of SA in physiological and pathological events in the brain.

In addition, SA-targeted biointerface materials modified with boronic acids small molecule or their derivatives were also presented in the form of three-dimensional particles. The size effect and luminescence properties from the modification or intrinsic characteristics of these particle materials make them highly suitable for SA and cancer cell detection [58,59]. For example, 4-mercaptophenylboronic acid functionalized gold nanoparticles as a simple and selective colorimetric sensor enabled the detection for SA with a shorter analysis time and a high accuracy, in virtue of color change of the solution form wine-red to blue after binding with SA [60]. In another similar work, fluorescent nanoparticles modified with aminophenylboronic acid achieved the capture and imaging of breast cancer cells with a higher binding efficiency and little cell toxicity [61]. Besides, Chaudhary et al. have reported a delicate SA biosensor by incorporating PBA group into arginine-containing short peptides. This biosensor clearly showed the excellent recognition capacity for SA on cell surface by combining selective binding of PBA with diol and electrostatic interactions between positively charged arginine and carboxylic acid residues of SA [62]. Therefore, these SA-targeted particle materials may serve as a favorable starting point for the development of devices for early cancer diagnosis, and of drug delivery systems for high accumulation in tumors. 

## 4. Polymeric Nanocarriers for Drug Delivery

Since the treatment efficacy of therapeutics is closely related to the site of drug action, persistent efforts have been dedicated to the development of advanced materials to achieve controllable drug delivery and precise drug release [63,64]. The aberrantly elevated sialylation on the cell membrane is an attractive target for cancer diagnosis and therapy. Hence, the surface functionalization of nanocarriers with SA-targeted ligands can achieve cancer cell selectivity and superior drug delivery for precision therapeutics. 

**Figure 6:**
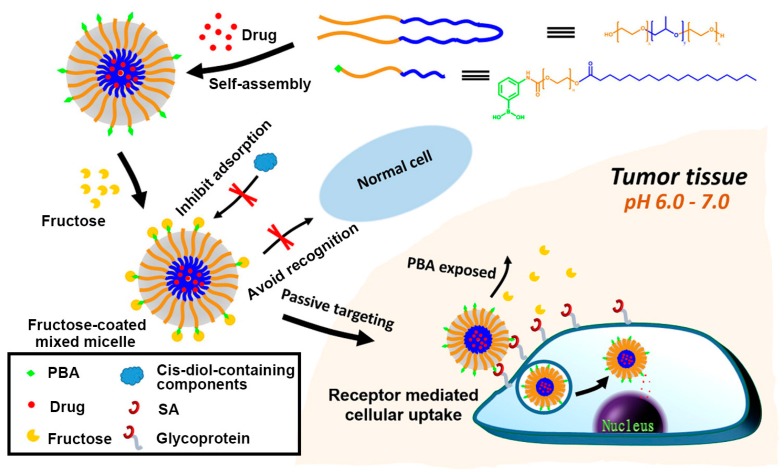
Illustration of pH-dependent targeting drug delivery system based on phenylboronic acid (PBA)-terminated micelle coated with fructose by selective binding of SA overexpressed on the cancer cell surface. Adapted with permission [66]. Copyright 2016, ACS.

As stated above, PBA and derivatives can specifically recognize SA overexpressed on the surface of cancer cell under the physiological conditions. Additionally, several advantages of PBA, such as nontoxicity and non-immunogenicity, make it an ideal ligand for targeting cancer cells. In 2013, Deshayes et al. developed PBA functionalized micellar nanocarriers incorporated with anticancer drugs for targeting SA on cancer cells for the treatment of solid tumors [65]. The PBA group was grafted onto poly(ethylene glycol)-*b*-poly(l-glutamic acid) to generate the block copolymer PBA-PEG-*b*-PLGA by one-pot reductive amination, the p*K*_a_ value of which was determined to be 9.7 through fluorescence titration. This guaranteed the recognition specificity toward SA at the physiological pH or even at slightly acidic intratumoral conditions in the trigonal form of PBA-SA complexation. After the anticancer drugs were loaded, the self-assembled micellar nanocarrier exhibited a faster cellular uptake than did micellar without PBA in vitro, as well as a prolonged blood circulation time and an improved retention time of micelles at the tumor site, which significantly enhanced the in vivo antitumor activity of the drugs. In order to further accurately target tumors and eliminate the side effect from the nonspecific binding of the PBA ligand to normal cells, Zhao et al. developed a similar PBA-terminated block copolymer micelle and creatively introduced fructose as an invisible cloak of the micelle nanocarrier for normal cells [66]. As shown in Figure 6, the micelles were first prepared by the self-assembly of the PBA-functionalized diblock polymer (PBA-PEG-C_18_) mixed with Pluronic P_123_, accompanied with the encapsulation of the hydrophobic antitumor drug doxorubicin (DOX). As PBA can bind to fructose more strongly than to other sugars in a physiological environment (pH 7.4), introduced fructose molecules prevent PBA on micelles from being recognized by other proteins or cells, leading to higher stability. However, under acidic intratumoral conditions (pH 6.5), unstable fructose-PBA complexation was replaced by SA-PBA complexation, achieving the goal of precise recognition of tumors. Specifically, in vitro uptake and cytotoxicity studies showed that fructose-coated micelle could reduce undesired cytotoxicity on normal cells and enhance the accumulation and cytotoxicity of DOX-loaded micelles in human hepatocellular liver carcinoma cells (HepG2). As a result, this simple decorating strategy may facilitate the development of PBA-targeted nanocarriers for highly tumor-specific drug delivery.

**Figure 7:**
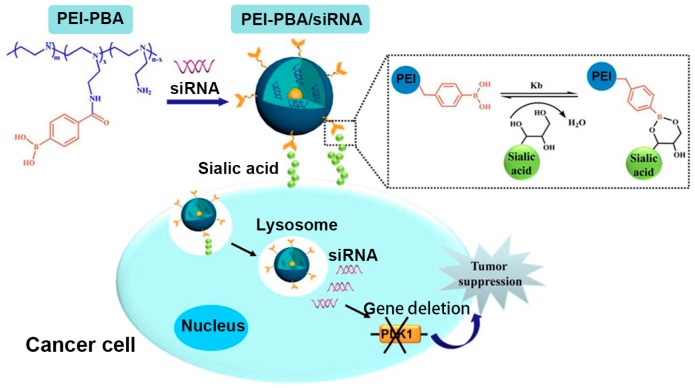
Novel siRNA delivery nanocarrier based on polyethylenimine-phenylboronic acid (PEI-PBA) conjugates for SA-targeted cancer therapy. Adapted with permission [69]. Copyright 2016, ACS.

In addition to chemotherapy based on antitumor drugs, inhibition of suitable target gene expression by small interference RNA (siRNA)-based gene therapy has opened up new possibilities for cancer treatment. Similarly, achieving the specific delivery and the enhanced cellular uptake of siRNA remains a challenging task. To this end, nanocarriers that can protect siRNA from being degraded and enable it to reach the tumor sites have naturally attracted the interest of researchers [67,68]. Ji et al. recently reported a novel siRNA delivery system based on polyethylenimine (PEI), a promising gene transfection vector, by combining the modification of PBA groups [69]. As shown in Figure 7, branched low-molecular-weight PEI was chosen as a less toxic main framework and the PBA group served as the specific ligand toward SA overexpressed on the cancer cell surface, both of which were integrated into an amphiphilic polymer PEI-PBA with capability of spontaneously self-assembling into a nanocarrier of siRNA. This PEI-PBA nanocarrier loaded with siRNA exhibited high biocompatibility, serum stability and RNase resistance; by contrast, it significantly increased the uptake of siRNA by cancer cells through the specific interaction between PBA and SA on the cancer cell surface. With these advantages, this siRNA delivery system successfully reduced the expression of the target gene and effectively inhibited tumor growth without significant toxicity in vivo. 

Although SAs play a vital role in many biologic processes, high sialylation, however, has been shown to contribute to cancer cell progression and tumor metastasis. For example, high-expressed sialoglycans on cancer cell surface have been proven to facilitate cancer cell detachment, protect from apoptosis and enhance migration and tissue invasion [70,71]. Accordingly, effective strategy to interference with SAs expression might provide an alternative and promising approach to prevent cancer metastasis. In 2013, Büll et al. explored the interference potential of a previously developed fluorinated SA analog (P-3Fax-Neu5Ac) in murine melanoma cells, and the results showed that this glycomimetic could effectively inhibit sialylation without affecting cell viability or proliferation, even in the presence of high concentrations of competing sialyltransferase [72]. Subsequently, they encapsulated P-3Fax-Neu5Ac into biodegradable poly(lactic-*co*-glycolic acid) (PLGA)-based nanoparticles coated with antityrosinase-related protein-1 antibody (anti-TRP-1) to further assess the inhibitory effect for cancer metastasis [73]. This tumor-targeted polymeric nanoparticles enabled the targeted delivery of tumor inhibitor, long-lasting SA expression blockade, and prevention for metastatic spread of melanoma cells in vivo. This specific and safe targeting to tumor cells to prevent metastatic metastasis offer an attractive approach to prevent tumor progression and metastasis, and even cure cancer.

## 5. Bio-Inspired Materials for SA Derivatives Separation/Enrichment

Creating new materials using bio-inspired strategies is one of the major challenges in materials science [74]. Biological systems that interact with or adapt to varied surrounding conditions by taking advantages of cooperative multiple non-covalent interactions have provided inspiration. Among which, carbohydrate to carbohydrate interactions (CCIs) have been revealed to play important roles in various cellular processes, such as cell adhesion, recognition and signaling [75,76]. For example, oligosaccharide Lewis X–Lewis X interaction induces cell adhesion in embryonic development [77] the ganglioside GM3 inhibit the activation of the epidermal growth factor receptor (EGFR) through CCIs between GM3 and *N*-glycan of EGFR [78]; both of these phenomena indicate that SAs as terminal saccharides of cellular surface glycoconjugates are involved in these cellular processes. Consequently, these features inspire researchers to develop novel material systems containing SA-targeted carbohydrate-based ligands based on CCIs.

**Figure 8:**
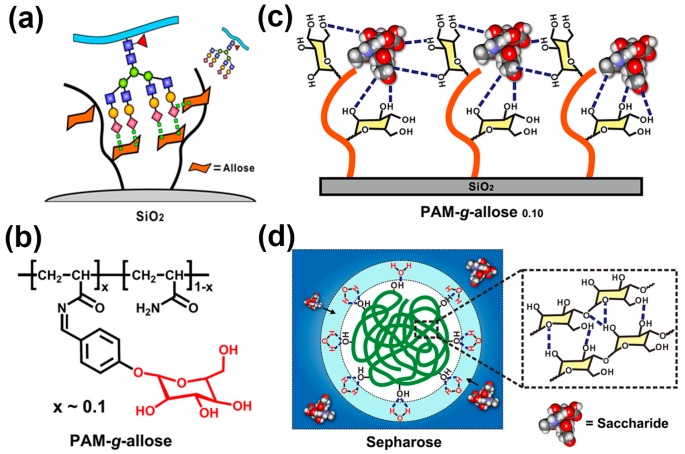
(**a**) Novel bio-inspired polymeric interface material for the separation and enrichment of SA-containing glycopeptides; (**b**) Chemical structure of the polymer PAM-*g*-allose; (**c**) Relaxed conformation and SA-binding models of the polymeric PAM-*g*-allose_0.10_. Hydrogen bonds are indicated by black dashed lines; (**d**) Illustration of the retention mechanism for Sepharose material, in which the hydrophilic water-rich layer is shown as a light blue circle. Adapted with permission [79]. Copyright 2016, ACS.

Since the determination of the significance of SA derivatives related to a wide variety of physiological and pathological processes, researchers have never ceased to pursue high-efficiency materials for the separation or enrichment of SA derivatives, especially sialylated glycopeptides. In this respect, our group creatively introduced a monosaccharide-based SA ligand into a polymeric interface material for sialylated glycopetide separation and enrichment, as shown in Figure 8a [79]. After the affinity screening of different monosaccharides by using fluorescent titration measurements and quartz crystal microbalance adsorption experiments, the monosaccharide allose was found to be capable of binding *N*-acetylneuraminic acid (Neu5Ac, a typical SA) through multiple hydrogen bond interactions with pH sensitivity. Then we integrated the allose into a polyacrylamide chain, obtained a glycopolymer (denoted as PAM-*g*-allose, Figure 8b) which significantly increased the binding sites of SA. Taking advantage of a favorable polymer conformation as well as pH-mediated binding interactions between allose and SA, this saccharide-responsive glycopolymer material exhibited high recognition specificity toward SA (Figure 8c). In contrast to conventional hydrophilic enrichment materials (e.g., Sepharose), our glycopolymer materials were highly hydrophobic (surface contact angles were larger than 75°) and resolved a common problem encountered by hydrophilic enrichment materials, in which hydrophilic water-rich layers obstruct the direct contact between the materials and surrounding saccharide molecules (Figure 8d). With these features, this saccharide-responsive polymer PAM-*g*-allose grafted silica gels facilitated the high-efficiency enrichment of sialylated glycopeptides and could even capture trace sialylated glycopeptides from real HeLa S3 cell lysate. This saccharide-responsive glycopolymer material will satisfy the requirements of glycoproteome analysis and promote the discovery of more SA-containing glycosites as biomarkers that are closely related to cancers or Alzheimer’s disease.

**Figure 9:**
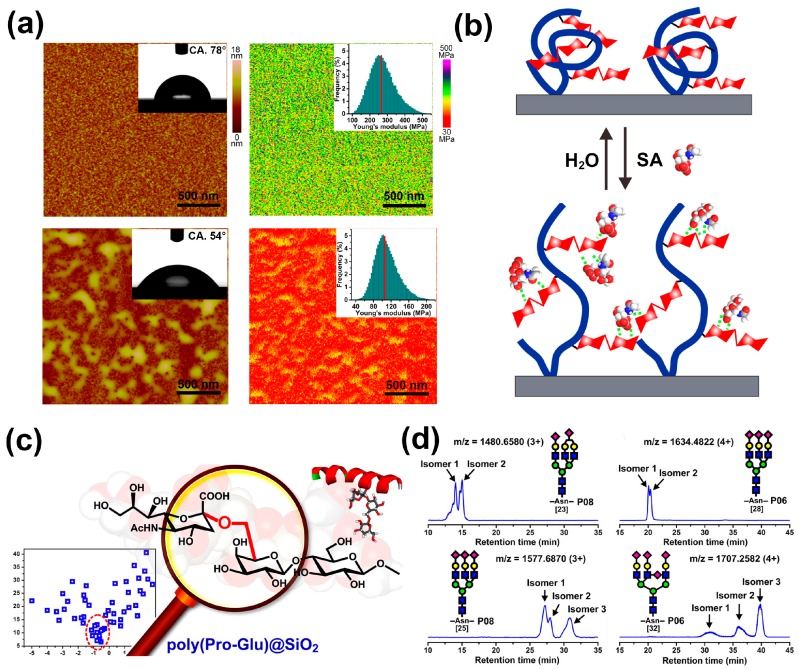
(**a**) SA-responsive remarkable changes in the surface topography, wettability, and stiffness of PAM-*g*-lactose-based interface material, upper panels show surface topography and contact angle (left) and surface Young’s modulus (right); lower panels show corresponding surface properties after treatment with SA; (**b**) SA recognition triggered conformational transition of the PAM-*g*-lactose polymer chains; (**c**) The screened dipeptide Pro-Glu by a series of orthogonal investigations for the recognition and discrimination of SA from other monosaccharides; (**d**) The polymer poly(Pro-Glu) displays accurate discrimination of glycosidic linkage isomers of oligosaccharides, as shown in typical extracted ion chromatograms, and each SA-containing glycopeptide splits into two or more peaks (corresponding to different glycosidic linkage isomers) on a poly(Pro-Glu)-based chromatographic column. Adapted with permission [85]. Copyright 2016, ACS.

Furthermore, in order to better mimic the structure of oligosaccharides involved in the SA recognition process, we recently improved the material design and focused on disaccharide to prepare a glycopolymeric interface material based on polyacrylamide grafted with lactose (denoted as PAM-*g*-lactose), with the expectation to present more outstanding target recognition and binding capability [80]. After similar screening steps, lactose came to the fore and specifically recognized SA. Moreover, the prepared interface material by the immobilization of PAM-*g*-lactose on a silicon wafer exhibited excellent SA-responsive specialties, which were reflected in the significant reversible changes of several surface macroscopic properties, such as surface topography, wettability, and stiffness (Figure 9a). The detailed investigation indicated that the distinct CCI between lactose and target SA led to the adsorption of SA and then triggered conformational transition of flexible chains through a destruction and reorganization process (Figure 9b), accompanied by dramatic changes in surface macroscopic properties. These features also enabled the polymer PAM-*g*-lactose grafted silica gels to achieve highly selective enrichment for SA-containing glycopeptides with a strong anti-interference level and high adsorption capacity.

Likewise, biomolecular recognition involving carbohydrate-protein interactions is ubiquitous in the biological system and also important to numerous fundamental biological processes. In this regard, lectins, as a type of saccharide-binding proteins, are widely used to recover SA-containing glycoproteins and neutral glycopeptides that carry specific carbohydrate motifs through affinity binding [81,82]. However, the limitation of affinity toward a narrow subset of the glycan structure and the higher cost of lectins make their application in large-scale SA-containing glycopeptides purification unattractive [83]. Inspired by the binding of lectins to saccharides through multiple hydrogen bonds from particular amino acid residues [84], our research group developed a novel dipepdide-based homopolymer, presenting a simple method of mimicking lectin [85]. After the optimization and screening of numerous dipeptide sequences through association constant-hydropathy index orthogonal investigations, several dipeptides (namely Pro-Glu, Pro-Asp, and Tyr-Glu) showed much higher binding affinity to SA than other monosaccharides, and higher discrimination capabilities than the other dipeptides. Next, we synthesized Pro-Glu homopolymers from propylene-acrylated monomers, which were coated on porous silica surfaces to generate the stationary phase material poly(Pro-Glu)@SiO_2_ (Figure 9c). This polymer material exhibited high-efficiency chromatographic separation toward SA and could accurately discriminate Neu5Ac and other SA with different chemical compositions or different linkage isomers. Moreover, the homopolymers displayed excellent performance in SA-containing glycopeptide separation and could even discriminate their subtly variable glycan structures, especially, isomeric glycosidic linkages (Figure 9d).

As a benefit from specific binding and precise discrimination toward SA-containing glycopetides of bio-inspired interface materials, the selectivity of bio-devices for tumor cells may obtain remarkable improvement when bio-inspired interface materials and their design concepts are focused. Moreover, the precise profiling of SA-containing glycopeptides can also be beneficial for progressing our understanding of the role that SA plays in physiological and pathological events.

## 6. Conclusions and Outlooks

Given the crucial roles of SAs in physiological and pathological processes, studying the SA-recognition receptor and further developing related artificial materials appear essential and have attracted considerable scholarly attention. Among them, artificial biointerface materials containing the SA receptor with the capacity of targeting SAs through a specific interaction on the interface have shown a trend of rapid development. In this review article, we focused on the recent studies on SA-targeted biointerface materials as well as their specific bio-applications from several aspects. As SA moieties are generally present on the surface of cancer cells, SA-imprinted particles and boronic acid-modified surfaces have been reported to achieve fascinating bio-applications for the imaging and detection of various cancer cells. Furthermore, polymeric nanocarriers possessing an SA-targeted biointerface with a stable architecture outside the target cancer cell, but easy degradability inside the cancer cell, exhibited excellent performance in tumor-selective drug delivery. In addition, SA-responsive bio-inspired interface materials with high recognition specificity presented enormous potential for the separation or enrichment of SA derivatives, which created new possibilities for the discovery of more roles of SA and its derivatives, such as a novel biomarker for a certain tumor. 

Although increasing prospects in SA-targeted biointerface materials have been revealed, in our opinion, many challenges remain to be solved. For example, current researches for comparing the amount of SA on different cell surfaces are mainly based on qualitative analysis methods. To further facilitate precise diseases diagnose, effective quantitative analysis methods should be established for the accurate determination of SA content on cell surface. Besides, because a relatively low level of SA is expressed on normal cells surface, the undesired targeting effects of SA-targeted biointerface materials on normal cells could not be eliminated, potentially causing serious dysfunctional consequences. To date, some types of artificial materials have also been developed and applied to detect SA in a living cells environment. However, the complicated sensing mechanism and unsatisfactory biocompatibility still restrict the suitability of SA monitoring in vivo. Therefore, future efforts for SA-targeted biointerface materials should be mainly devoted to the following several aspects. The first point is to improve the targeting accuracy of SA-targeted biointerface materials, which will revolutionize the therapy of various cancers by means of spatially and temporally controlled anti-tumor drug delivery. For example, a prior study found that the introduction of fructose as a shield of a PBA-functionalized nanocarrier to prevent the undesired recognition from normal cells provided a new insight into increasing target specificity [66]. In addition, another central task for SA-targeted biointerface material design is to improve the sensitivity and biocompatibility in vivo. In this regard, creating a new material system by mimicking the natural biological system represents a promising solution. For example, future efforts should be made to explore the structure of lectin in detail, screen for suitable oligopeptide fragments and further develop oligopeptide or even glycopeptide-based polymeric materials capable of specifically recognizing SA with high sensitivity and satisfactory biocompatibility. Moreover, the SA recognition of SA-targeted biointerface materials is usually the first step, and transforming this recognition signal into the easily detected changes in material properties (e.g., visible color changes) remains a major challenging but an intriguing project for SA-targeted biointerface materials. All these challenges and opportunities make SA-targeted biointerface materials a fertile ground for the close cooperation of material scientists, chemists and biologists interested in disclosing more complete understanding on SA and developing more effective approaches for the diagnosis and therapy of SA-associated diseases.
